# Supplementary material for: Effects of Different Ratios of Carbohydrate–Fat in Enteral Nutrition on Metabolic Pattern and Organ Damage in Burned Rats
Source: Nutrients. 2022 Sep 4;14(17):3653. doi: 10.3390/nu14173653 (PMC9460118; doi:10.3390/nu14173653)
Supplement: Supplementary file 1 [file nutrients-14-03653-s001.zip › nutrients-1869135-supplementary.pdf]

Supporting Information

Effects of different ratios of carbohydrate–fat in enteral nutrition on metabolic pattern and organ damage in burned rats

Yongjun Yang [1,†], Sen Su [1,†], Yong Zhang [2,†], Dan Wu [1], Chao Wang [2], Yan Wei [1] and Xi Peng [1,2,3,*]

[1] Clinical Medical Research Center, Southwest Hospital, Third Military Medical University (Army Medical University), Chongqing 400038, China
[2] State Key Laboratory of Trauma, Burns and Combined Injury, Institute of Burn Research, Southwest Hospital, Third Military Medical University (Army Medical University), Chongqing 400038, China
[3] Shriners Burns Hospital, Massachusetts General Hospital, Harvard Medical School, Boston, MA 02114, USA
* Correspondence: pxlrmm@163.com
† These authors contributed equally to this work.

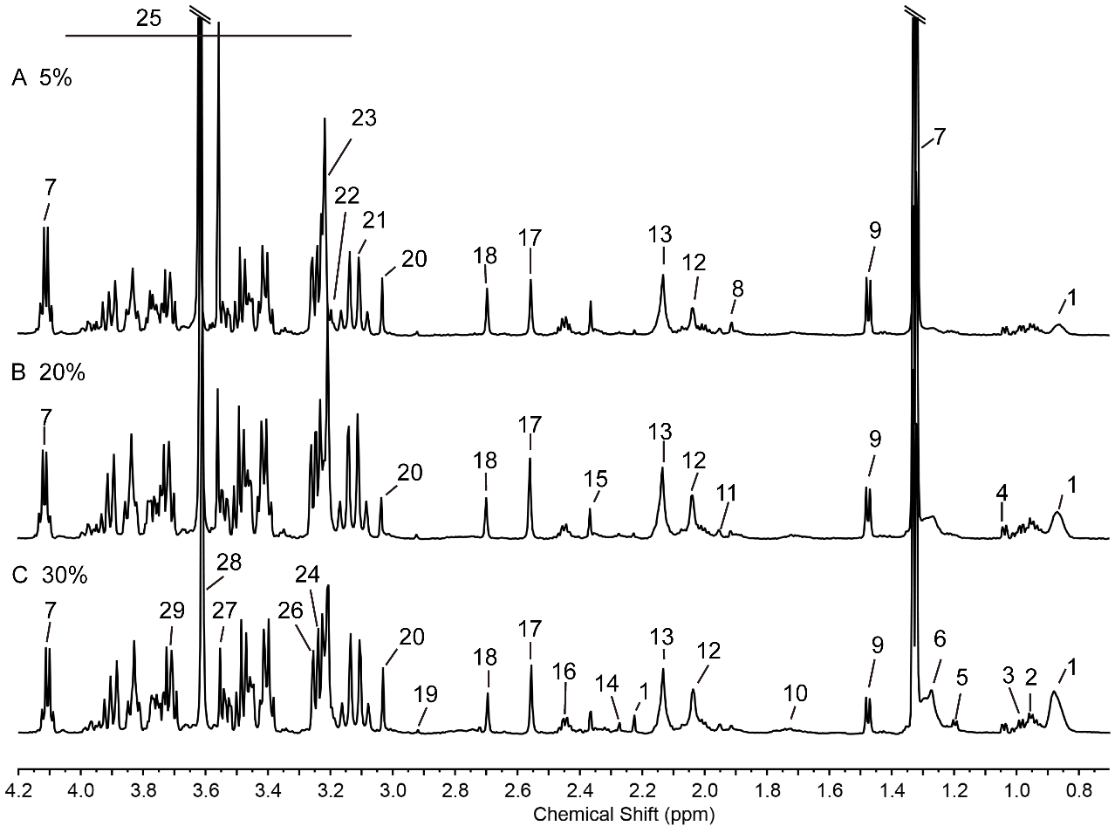

Figure S1. Representative 600 MHz ^{1}H NMR spectra of plasma (A. 5%, B. 20% and C. 30% group). The ^{1}H-NMR signals identified were: 1. Lipid, 2. Isoleucine, 3. Leucine, 4. Valine, 5. Isobutyrate, 6. 3-hydroxybutyrate, 7. Lactate, 8. Lysine, 9. Alanine, 10. Arginine, 11. Acetate, 12. N-acetylated glycoproteins (NAG), 13. O-acetylated glycoproteins (OAG), 14. Acetoacetate, 15. Pyruvate, 16. Glutamine, 17. Succinate, 18. Dimethylamine, 19. Dimethylglycine, 20. Creatinine, 21. Phenylalanine, 22. Choline, 23. Trimethylamine N-oxide (TMAO), 24. Phosphocholine, 25. α-glucose, 26. Betaine, 27. Glycine, 28. Oxalic acid, and 29. Glycerol.

Table S1. Nutritional supplement formula.

	Control group		Burn + 5% fat group		Burn + 10% fat group		Burn + 20% fat group		Burn + 30% fat group	
**Name**	**Weight**	**Energy**	**Weight**	**Energy**	**Weight**	**Energy**	**Weight**	**Energy**	**Weight**	**Energy**
Peptisorb	100 g	402 Kcal	100 g	402 Kcal	100 g	402 Kcal	100 g	402 Kcal	100 g	402 Kcal
Glucose	-	-	155.8 g	623.1 Kcal	104.7 g	562.8 Kcal	100.5 g	442.2 Kcal	-	-
Amino acid	28.14 g	112.56 Kcal	43.28 g	180.9 Kcal	43.28 g	180.9 Kcal	43.28 g	180.9 Kcal	28.14 g	112.56 Kcal
Medium long chain Fatty acid emulsion	115.64 ML	168.84 Kcal	-	-	41.3 ML	60.3 Kcal	123.9 ML	180.9 Kcal	115.64 ML	168.84 Kcal
Total	272.34 g	683.4 Kcal	299.08 g	1206 Kcal	301.42 g	1266.3 Kcal	398.28 g	1206 Kcal	272.34 g	683.4 Kcal

Table S2. Assay values of REE in each group of rats from 1–14 days after burn injury.

		PBD0	PBD1	PBD2	PBD3	PBD4	PBD5	PBD6	PBD7	PBD8	PBD9	PBD10	PBD11	PBD12	PBD13	PBD14
REE (Kcal/kg/d)	C	251.5±11.2	245.9±11.7	246.4±12.8	249.8±9.9	244.4±9.9	249.8±10	253.7±13	251.3±11.5	244.8±9.9	248.4±12.6	251.7±12.8	252.8±12.1	252.9±11.9	249.1±14.5	251.4±12.6
	5 %	256.8±14	246.9±11.8	250.3±5	234.6±17.2	238.5±12.9	247.1±11.2	256.7±5.2	255.4±14.3	263.4±12.9	261.5±12.1	262±6.2	259.3±14.6	263.6±7.3	268.4±10.4	267.8±12.6
	10%	259.1±16	242.7±9.9	240.9±20.7	223.5±18.7	230.1±13.5	238.1±12.6	244±14.1	243.2±10.2	244.6±11.2	242±12.4	248.3±10	245.5±11.7	243.3±8.8	249.7±11.6	255.5±12.3
	20%	253.6±11.5	230.8±18.5	225.1±15.2	211.9±11.4	225±16.7	234.6±11.1	241.2±12.7	250.1±15.6	250.7±10.7	269.6±11.4	269.9±12	273.2±11.1	273±17.5	273±13	276.8±10.2
	30%	259.4±10.1	222.2±10.8	214±12.6	197.3±11.5	218.4±12.4	227.3±11.6	255.2±11.2	269.7±14.8	280.3±12	278.9±13.8	276.8±12.8	281.6±11.8	277.6±13.4	279.3±12.5	289±10.4

Table S3. The actual amount of energy supplied to each group of rats from 1–14 days after burn injury.

		PBD0	PBD1	PBD2	PBD3	PBD4	PBD5	PBD6	PBD7	PBD8	PBD9	PBD10	PBD11	PBD12	PBD13	PBD14
Energy supply (Kcal/kg/d)	C	251.7±11.8	245.4±11.4	246.3±12.7	249.3±9.5	244.2±9.2	249.3±9.4	253.7±13.6	251.5±11.4	244.1±9.2	248.2±12.2	251.4±12.5	252.5±12.6	252.1±11.2	248.4±14.7	251.5±11.3
	5 %	193.1±10.5	185.7±8.81	187.1±3.3	175.4±123	178.1±9.5	185.2±8.5	192.2±4.2	191.2±10.2	263.7±12.9	261.4±12.3	262.1±6.8	259.1±14.4	263.2±7.1	268.3±10.2	267.9±12.1
	10%	194.2±12.6	182.2±7.1	180.1±15.7	167.3±14.7	172.3±10.7	178.4±9.2	183.8±10.2	182.3±7.7	244.8±11.9	242.8±12.4	248.5±10.8	245.5±11.7	243.5±8.2	249.5±11.4	255.2±12.9
	20%	190.5±8.4	172.7±13.5	168.1±11.0	158.2±8.2	168.1±12.2	175.7±8.2	180.1±9.2	187.2±11.3	250.4±10.8	269.2±11.8	269.3±12.8	273.2±11.1	273.6±17.8	273.1±12.4	276.4±10.3
	30%	193.9±7.7	166. 5±8.6	160.2±9.2	147.8±8.1	163.2±9.5	170.3±8.6	191.7±8.1	202.6±11.8	280.4±12.7	278.1±12.9	276.4±12.1	281.2±11.4	277.5±13.3	279.1±12.6	289±10.6

Table S4. ^{1}H-NMR characteristic signals of the identified metabolites in serum.

ID	Metabolites	^{1}H Shift(δ)
**1**	Lipid	0.891(t) 1.210(m) 1.221(m) 1.232(m) 1.590(m) 2.018(m) 2.238(m) 2.742(m) 2.749(m) 2.761(m)
**2**	Isoleucine	0.943(t) 1.000(d) 1.008(d) 1.284(m) 1.459(m) 1.961(m)
**3**	Leucine	0.955(d) 0.965(d) 0.975(d) 1.691(m) 1.707(m) 3.685(dd) 3.753(d)
**4**	Valine	0.988(d) 1.020(d) 1.040(d) 1.052(d) 2.285(m) 3.570(d) 3.617(d)
**5**	Isobutyrate	1.361(d)
**6**	3-hydroxybutyrate	1.200(d) 2.293(m) 2.380(m) 4.131(m)
**7**	Lactate	1.341(d) 4.108(q)
**8**	Lysine	1.434(m) 1.689(m) 1.719(m) 1.886(m) 1.897(m) 3.031(t) 3.767(t)
**9**	Alanine	1.480(d) 1.492(m) 3.783(q)
**10**	Arginine	1.681(m) 1.730(m) 1.926(m) 3.257(t) 3.774(m)
**11**	Acetate	1.914(s)
**12**	N-acetylated glycoproteins (NAG)	2.04 (s)
**13**	O-acetylated glycoproteins (OAG)	2.14 (s)
**14**	Acetoacetate	2.273(s) 3.441(s)
**15**	Pyruvate	2.318(s) 2.372(s)
**16**	Glutamine	2.14(m) 2.46(m) 3.77(t)
**17**	Succinate	2.395(s)
**18**	Dimethylamine	2.71(s)
**19**	Dimethylglycine	2.930(s) 3.723(s)
**20**	Creatinine	3.938(s) 3.051(s) 4.066(s)
**21**	Phenylalanine	3.119(dd) 3.260(dd) 3.962(dd) 3.991(dd)
**22**	Choline	3.208(s) 3.657(m) 4.072(m)
**23**	Trimethylamine N-oxide (TMAO)	3.21(s)
**24**	Phosphocholine	3.218(s) 3.585(m) 4.142(m)
**25**	α-glucose	3.429(t) 3.542(dd) 3.708(t) 3.732(dd) 3.822(dd) 3.840(dd)
**26**	Betaine	3.271(s) 3.915(s)
**27**	Glycine	3.558(s)
**28**	Oxalic acid	3.621(s)
**29**	Glycerol	3.552(dd) 3.649(dd) 3.795(m)
**30**	Triglycerides	4.072(m)

Type of ^{1}H Shift(δ): s, single; d, doublet; t, triplet; m, multiple; q, quartet; dd, doublet of doublets.

Table S5. Identified metabolites from different groups with S-plot, VIP and P-value.

Metabolites	VIP	*P*-value*
**Lipid**	4.10675	< 0.0001
**Isoleucine**	1.65065	0.0386
**Leucine**	1.6876	0.0045
**3-hydroxybutyrate**	2.95366	< 0.0001
**Lactate**	9.21987	0.0047
**Alanine**	6.30719	0.0008
**Pyruvate**	4.26619	0.0012
**Dimethylamine**	2.25777	< 0.0001
**Betaine**	2.99439	0.0001
**Glycine**	8.23387	< 0.0001
**Oxalic acid**	2.28375	0.0392
**Glutamine**	2.23501	0.0441

* One-way ANOVA for comparisons between multiple groups

Table S6. The associated metabolic pathways of the differential metabolites in Table S5.

**Pathway Name**	**Hits/Total**	***p*-value**	**FDR**	**–log(p)**	**Impact**
**Alanine, aspartate and glutamate metabolism**	3/28	0.00113	0.034	2.95	0.11378
**Glyoxylate and dicarboxylate metabolism**	3/32	0.00169	0.034	2.77	0.10582
**Glycine, serine and threonine metabolism**	3/34	0.00201	0.034	2.70	0.31772
**Pyruvate metabolism**	2/22	0.01231	0.172	1.91	0.20684
**Glycolysis / Gluconeogenesis**	2/26	0.01702	0.204	1.77	0.10044
